# Temporal expression patterns of insulin-like growth factor binding protein-4 in the embryonic and postnatal rat brain

**DOI:** 10.1186/1471-2202-14-132

**Published:** 2013-10-31

**Authors:** Xiaohua Jiang, Junpeng Zhao, Lili Ju, Yujun Liu, Beibei Wang, Xifeng Zou, Changlei Xu

**Affiliations:** 1 Beijing Institute for Brain Disorders, Beijing Centre for Neural Regeneration and Repair, Beijing Key Laboratory of Brain Major Disorders, Key Laboratory for Neurodegenerative Diseases of The Ministry of Education, Capital Medical University, Beijing, 100069, P. R. China

**Keywords:** Insulin-like growth factor binding protein-4 (IGFBP-4), Rat, Embryonic brain, Postnatal brain, Development

## Abstract

**Background:**

IGFBP-4 has been considered as a factor involving in development of the central nervous system (CNS), but its role needs to be further clarified. In present study, the localization of IGFBP-4 expression in the embryonic forebrain, midbrain and hindbrain was determined using immunohistochemistry, and the levels of IGFBP-4 protein and mRNA were semi-quantified using RT-PCR and Western blot in the embryonic (forebrain, midbrain and hindbrain) and postnatal brain (cerebral cortex, cerebellum and midbrain).

**Results:**

A clear immunoreactivity of IGFBP-4 covered almost the entire embryonic brain (forebrain, midbrain, hindbrain) from E10.5 to E18.5, except for the area near the ventricle from E14.5. The change of IGFBP-4 mRNA level was regularly from E10.5 to E18.5: its expression peaked at E13.5 and E14.5, followed by gradual decreasing from E15.5. The expression of IGFBP-4 protein was similar to that of mRNA in embryonic stage. After birth, the pattern of IGFBP-4 expression was shown to be rather divergent in different brain areas. In the cerebral cortex, the IGFBP-4 mRNA increased gradually after birth (P0), while the protein showed little changes from P0 to P28, but decreased significantly at P70. In the cerebellum, the IGFBP-4 mRNA decreased gradually from P0, reached the lowest level at P21, and then increased again. However, its protein level gradually increased from P0 to P70. In the midbrain, the IGFBP-4 mRNA first decreased and reached its lowest level at P28 before it increased, while the protein remained constant from P0 to P70. At P7, P14, P21, P28 and P70, the levels of IGFBP-4 mRNA in the cerebral cortex were significantly higher than that in the cerebellum or in the midbrain. Differently, the protein levels in the cerebellum were significantly higher than that either in the cerebral cortex or in the midbrain at P14, P21, P28 and P70.

**Conclusions:**

The temporal expression pattern of IGFBP-4 in the embryonic brain from E10.5 to E18.5 was consistent with the course of neurogenesis in the ventricular zone, suggesting an important role of IGFBP-4 in regulating differentiation of neural stem cells. A strikingly higher abundance of the IGFBP-4 protein observed in the cerebellum from P14 to P70 suggests that IGFBP-4 may participate in the maintenance of cerebellar plasticity.

## Background

The development of the mammalian central nervous system (CNS) is a remarkable phenomenon. Since the CNS is composed of millions of distinct neural cells, its complex and accurate functions must depend on the highly organized architecture of the cells that assemble in precise circuits. These distinct classes of cells composing the functional networks, are positioned at specific coordinates, in a precise number, with a spatial and temporal hierarchy. Researchers working in developmental neuroscience are still seeking to answer certain questions involving the control of diversity, migration, and connection of neural cells [1]. Following this direction, investigators believe that the way to understand these mechanisms requires the analysis of differential gene expression in different developmental stages of the brain.

Among the large number of differential genes identified as important factors for regulating brain development at certain stages, we have found that the gene for insulin-like growth factor binding protein-4 (IGFBP-4) is expressed more highly in mature neurons than in neural precursors, indicating that IGFBP-4 may potentially act as a proneuronal differentiation factor [2]. It is already known that IGFBP-4 is one member of the IGFBP family, which is mainly composed of six highly homologous proteins that bind insulin-like growth factors (IGFs) with high affinity to regulate its activity [3].

IGFBP-4 is the smallest IGFBP and is unique in having two extra Cys residues in the variable L-domain encoded by exon 2 [4]. IGFBP-4 also contains an N-linked glycosylation site and commonly exists in biological fluids as a doublet: a 24-kDa nonglycosylated form and a 28-kDa glycosylated form [5]. Authentic rat IGFBP-4 was a mixture of about 20% glycosylated and 80% non-glycosylated forms, and the glycosylation of IGFBP-4 does not affect its binding to IGFs [6]. The physiological significance of the glycosylation in IGFBP-4 is unknown.

IGFBP-4 is an important physiological regulator of IGF actions in bone cells and other cell types as well [7,8]. IGFBP-4 usually inhibits IGFs effects, but IGFs can decreased IGFBP-4 levels by activating an IGFBP-4-specific protease-PAPP-A, so IGFBP-4 may act as a potent inhibitor of the anabolic effects of IGF-I or -II by regulating IGF bioavailability [9]. Previous data showed that IGFBP-4 is a pro- or anti-apoptotic factor that binds to an unknown membrane receptor and negatively or positively regulates apoptosis-induced factors. IGFBP-4 also may act by modulating the expression and secretion of other IGFBPs, such as IGFBP-3 and IGFBP-6 [10]. Although still preliminary, until now, IGFBP-4 is the only IGFBP that, when deleted, alters cell growth [11].

IGF-IGFBP system is already recognized as central to processes of cell growth, differentiation, and migration [9,12]. The clear importance of IGFs in the CNS development underscores the need for examining the expression and action of molecules, which are capable of regulating and mediating IGF activities. IGFBP-2, -4, and -5 are the most predominant IGFBPs in the brain [12]. In general, IGFBP-2 and -5 were detected in same cells, whereas IGFBP-4 and -5 were expressed mainly in different cells, which suggests IGFBP-4 may have a specific function [13].

In view of the predominantly local activity of various proteins, the distribution of expression of IGFBP-4 may have a particular significance.

In the rat embryos of embryonic day 14 (E14), IGFBP-4 transcripts are expressed widely in non-neural areas of the head [14]. In E15 rat embryos, IGFBP-4 transcripts are undetectable in other regions of brain except in the choroid plexus primordium and the meninges; the appearance of IGFBP-4 in brain parenchyma is observed from E20, and its expression is more widespread with increasing age [15]. IGFBP-4 was found in all gross anatomical divisions of rat brain from E15 until adulthood (at E15, E20, postnatal day 1 (P1), P5, Adult), and IGFBP-4 mRNA tends to be more abundant at the youngest ages [16].

In the mouse embryo, IGFBP-4 transcripts can be detected as early as E11 in different regions, including the telencephalon, mesencephalon, snout, tongue, and differentiating sclerotomes; its mRNA is undetected in the brain after E14, but clearly detectable in the lung, liver, kidneys, intestine, and vertebrae (from E11 to E18). At E18, IGFBP-4 transcripts cannot be detected in choroid plexus and meninges [13]. IGFBP-4 mRNA and protein are detected in the telencephalon, mesencephalon of E13.5 mouse embryos, and the consistence of localization patterns between IGFBP mRNA and protein may suggest that the IGFBP-4 functions in an autocrine or paracrine manner [17].

Previous findings suggest that IGFBP-4 expression may be regulated during brain development, but unfortunately, the precise contributions of IGFBP-4 to brain development are still not clear, since previous studies only selected limited time-points of IGFBP-4 temporal expression, and/or did not quantify IGFBP-4 expression. The present study therefore, aimed to examine precisely the temporal expression of IGFBP-4 at different developmental time-points in the rat brain, by using immunohistochemistry, quantitative real-time PCR, and Western blot. We hope this observation could provide a foundation for understanding the role of IGFBP-4 in brain development.

## Results

### Expression pattern of IGFBP-4 in the embryonic brain

**Figure 1:**
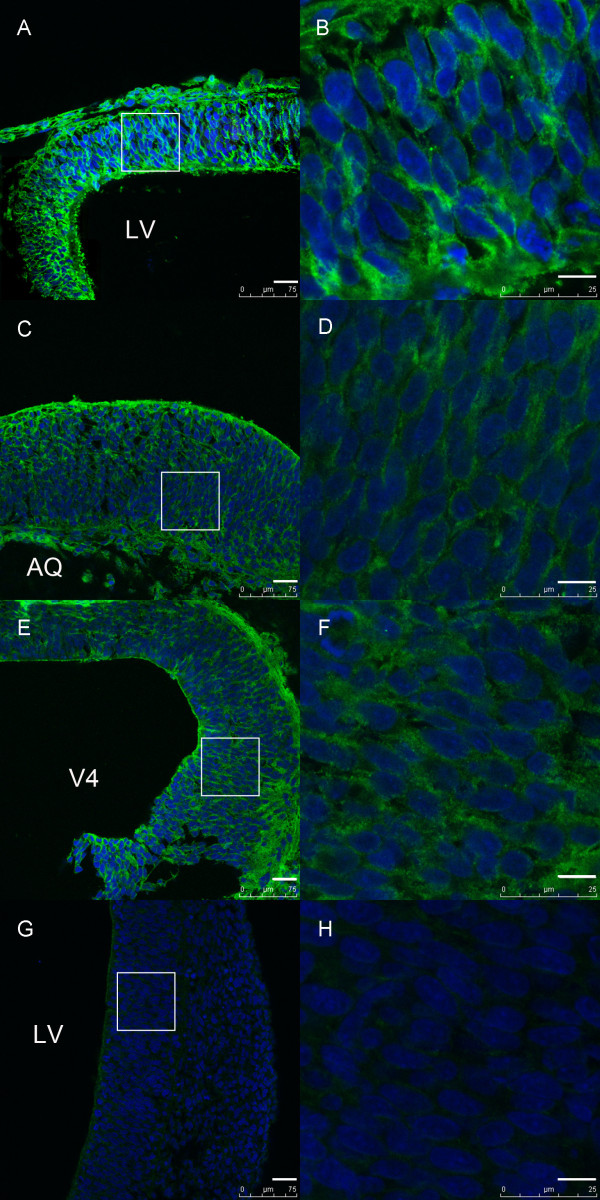
**Expression of IGFBP-4 protein at E13.5 by immunofluorescent staining. (A, B)** Strong positive signal for IGFBP-4 protein was detected in the forebrain. **(C, D)** In the midbrain, positive signal of IGFBP-4 protein was detected, but it was slightly weaker than in the forebrain. **(E, F)** In the hindbrain, the positive signal for IGFBP-4 protein was similar to that in the midbrain. **(G, H)** Negative control. LV-lateral ventricles, AQ-cerebral aqueduct, V4-fourth ventricle. Scale bar = 30 μm in **A,C,E,G**; 10 μm in **B,D,F,H**.

Immunofluorescent staining showed a clear immunoreactivity of IGFBP-4 in almost the whole embryonic brain, the forebrain (Figure 1A, B), midbrain (Figure 1C, D), hindbrain (Figure 1E, F), and in the meningeal cells, from E10.5 to E18.5, although a relatively higher level of IGFBP-4 expression was seen in the forebrain. The intensity of IGFBP-4 immunoreactivity was relatively stronger at E13.5 than that at other time-points (Figure 1). From E15.5, however, the intensity decreased gradually. Similar immunoreactivity distribution of IGFBP-4 in the brain was observed using goat anti- and rabbit anti-IGFBP4 antibody.

**Figure 2:**
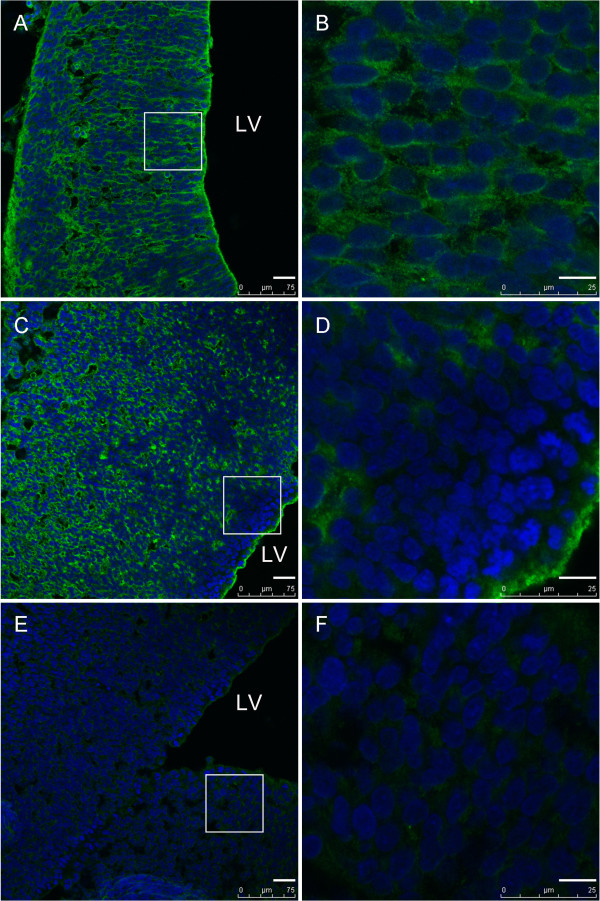
**The comparision of expression pattern of IGFBP-4 protein in the forebrain at E14.5 and E16.5. (A, B)** At day E14.5, the positive signal for IGFBP-4 protein was detectable widespread in brain regions including VZ. **(C, D)** At day E16.5, the positive signal for IGFBP-4 protein was still detectable widespread in brain regions, but fluorescent signals were not found at any significant level in cells near the ventricle. **(E, F)** Negative control. LV-lateral ventricles. Scale bar = 30 μm in **A,C,E**; 10 μm in **B,D,F**.

The expression pattern of IGFBP-4 changed from E14.5. More specifically, IGFBP-4 cannot be detectable in the ventricular zone at this stage, and the signal intensity displayed a gradient distribution in the lateral wall of the lateral ventricles (the higher the signal intensity, the further from the ventricular zone). At E16.5 the fluorescent intensity decreased significantly, although it was still detectable widely in brain regions (Figure 2). Fluorescent signals were not apparent in the cells near the ventricle at E16.5.

### IGFBP-4 mRNA level in rat embryonic brain

**Figure 3:**
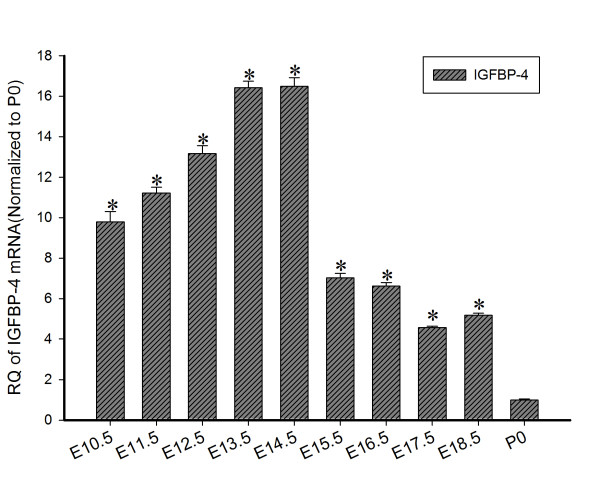
**Temporal expression of IGFBP-4 mRNA in embryonic rat brains.** The level of IGFBP-4 mRNA in embryonic brains changed from days E10.5 to E18.5 and reached a peak at days of E13.5 and E14.5. RQ of IGFBP-4 mRNA in the brains before birth was significantly higher than that at P0 (*P < 0.05).

Real-time PCR was used to analyze changes in mRNA level of IGFBP-4 in embryonic rat brain from E10.5 to E18.5 (Figure 3). The level of IGFBP-4 mRNA at E10.5 was 9.8 times that at P0 and then began to increase gradually and reached a peak at E13.5 and E14.5 (16.4 times and 16.5 times greater that at P0, respectively). The expression decreased gradually from E15.5, at E18.5 it was only 5 times that at P0.

### IGFBP-4 protein level in rat embryonic brain

**Figure 4:**
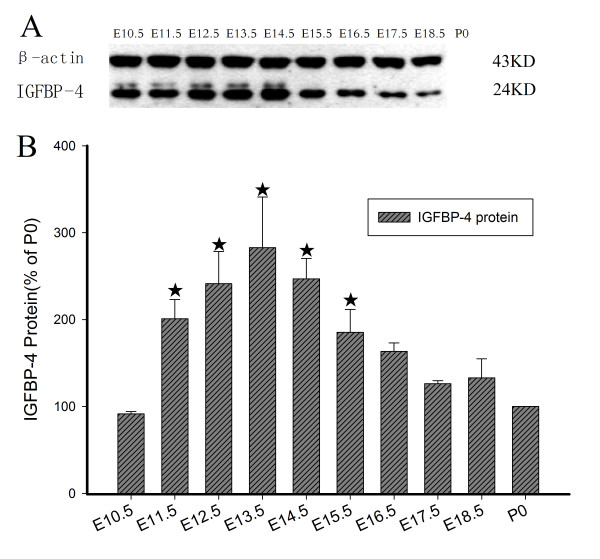
**Temporal expression of IGFBP-4 protein in embryonic rat brains. (A)** The results of Western blot, **(B)** A quantification of IGFBP-4 protein achieved by densitometry of 12 experiments. The expression pattern of IGFBP-4 protein is similar to that of IGFBP-4 mRNA. The expression peak was at E13.5 (282.75% of that at P0). ★- the level of IGFBP-4 protein is significantly higher than P0 (all *P* < 0.05).

The level of IGFBP-4 protein was determined by Western blotting, and the result was consistent with those by real-time PCR (Figure 4). It was shown that the protein level increased gradually from E10.5, and reached a peak at E13.5 (282.75% of that at P0). The level then decreased gradually from E14.5, and at E18.5 it was only 132.88% of P0. Glycosylated form of IGFBP-4 was detectable from E10.5 to E14.5, but not seen after E15.5.

### IGFBP-4 mRNA expression in postnatal rat brain

The expression of IGFBP-4 mRNA was analyzed in three areas of postnatal rat brain, cerebral cortex, cerebellum, and midbrain. ANOVA statistical analysis revealed significant differences in mRNA levels of IGFBP4 in each region of the brain at different time-points and in different regions at each time-point (data show in the Table 1).

**Table 1:** IGFBP-4 mRNA levels in the postnatal rat brains

**mRNA**	**F-cerebral cortex**	**F-cerebellum**	**F-midbrain**
P0	1.00000	1.01736 ± 0.002157	1.09569 ± 0.001673
P7	2.09334 ± .001591	1.07184 ± 0.000465	1.02553 ± 0.001133
P14	3.16452 ± .026631	0.45317 ± 0.000087	0.67977 ± 0.000694
P21	4.70771 ± .006930	0.40988 ± 0.000726	0.36422 ± 0.000309
P28	2.75328 ± .001084	0.63748 ± 0.001122	0.24572 ± 0.000182
P70	3.50929 ± .004576	1.85869 ± 0.001430	0.47534 ± 0.000835

**Figure 5:**
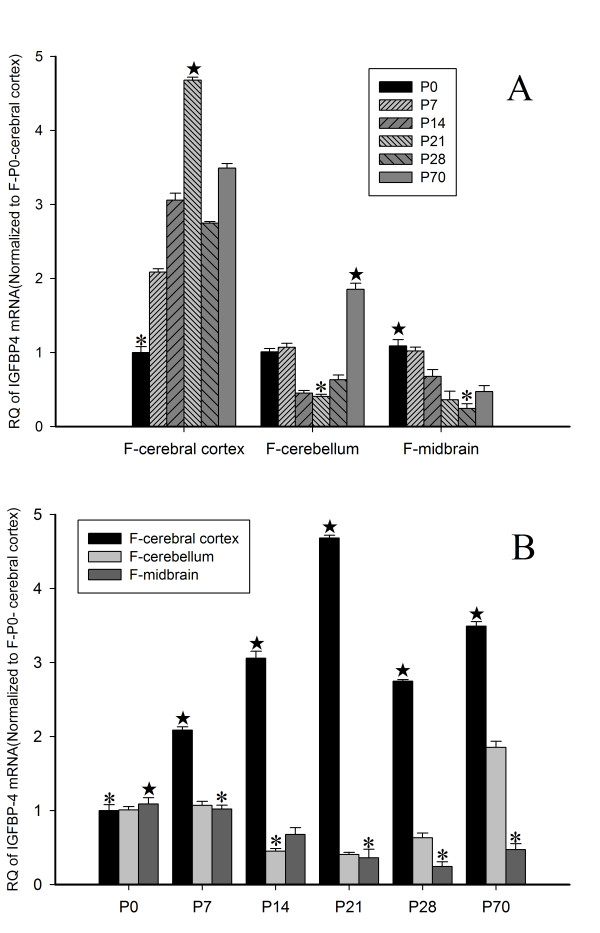
**IGFBP-4 mRNA expression in postnatal rat brains. (A)** The temporal expression of IGFBP-4 mRNA in specific brain regions. (In the cerebral cortex, *- the level of IGFBP-4 mRNA at P0 is significantly lower than at other time-points, ★- the level of IGFBP-4 mRNA at P21 is significantly higher than at other time-points; In the cerebellum, *- the level of IGFBP-4 mRNA at P21 is significantly lower than at other time-points, ★- the level of IGFBP-4 mRNA at P70 is significantly higher than at other time-points; In the midbrain, *- the level of IGFBP-4 mRNA at P28 is significantly lower than at other time-points, ★- the level of IGFBP-4 mRNA at P0 is significantly higher than at other time-points; all *P* < 0.05) **(B)** The spatial expression of IGFBP-4 mRNA at specific time-points. (At P0, *- the level of IGFBP-4 mRNA in cerebral cortex is significantly lower than in other brain regions; ★- IGFBP-4 mRNA in midbrain is significantly higher than in other brain regions. At P7, P21, P28 and P70, *- the level of IGFBP-4 mRNA in midbrain is significantly lower than in other brain regions; ★- IGFBP-4 mRNA in cerebral cortex is significantly higher than in other brain regions. At P14, *- the level of IGFBP-4 mRNA in cerebellum is significantly lower than in other brain regions; ★- IGFBP-4 mRNA in cerebral cortex is significantly higher than in other brain regions; all *P* < 0.05) (F-female rat).

In the cerebral cortex, the expression of IGFBP-4 mRNA increased gradually after birth (P0), and reached a peak at P21 (*p* < 0.05). Then it remained at a relatively high level till P70. It should be pointed out that the level at P21 was still lower than that in the embryo. In the cerebellum, IGFBP-4 mRNA level decreased gradually from P0, and reached the lowest level at P21, and then increased again and reached its highest level at P70 (*p* < 0.05). In the midbrain, the level of IGFBP-4 mRNA also decreased gradually from P0, and reached its lowest level at P28 (*p* < 0.05). It then increased and remained at a medium level at P70 (Figure 5A).

In order to emphasize the area specificity of IGFBP-4 mRNA expression in the brain, we further analyzed the differences between the cerebral cortex, the cerebellum and the midbrain at each time-point using the same data as shown in Figure 5A. At P0, the level of IGFBP-4 mRNA in the midbrain was higher than that in the cerebral cortex and cerebellum (*p* < 0.05, analysis results shown in Additional file 1). At P14, the level was highest in the cerebral cortex, moderate in the midbrain, and lowest in the cerebellum (*p* < 0.05). Afterwards, the levels of IGFBP-4 mRNA remained the highest in the cortex, and lowest in the midbrain at P21, P28, and P70 (*p* < 0.05), which were also seen at P7 (Figure 5B).

### IGFBP-4 protein level in postnatal rat brain

**Figure 6:**
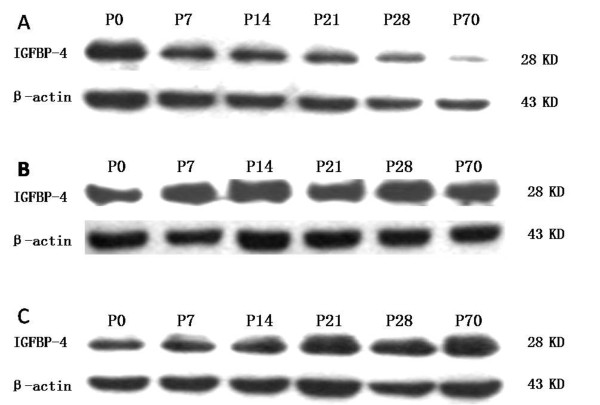
The results of Western blot: (A) cerebral cortex, (B) cerebellum, (C) midbrain.

IGFBP-4 protein was detected using Western Blot (Figure 6).

**Figure 7:**
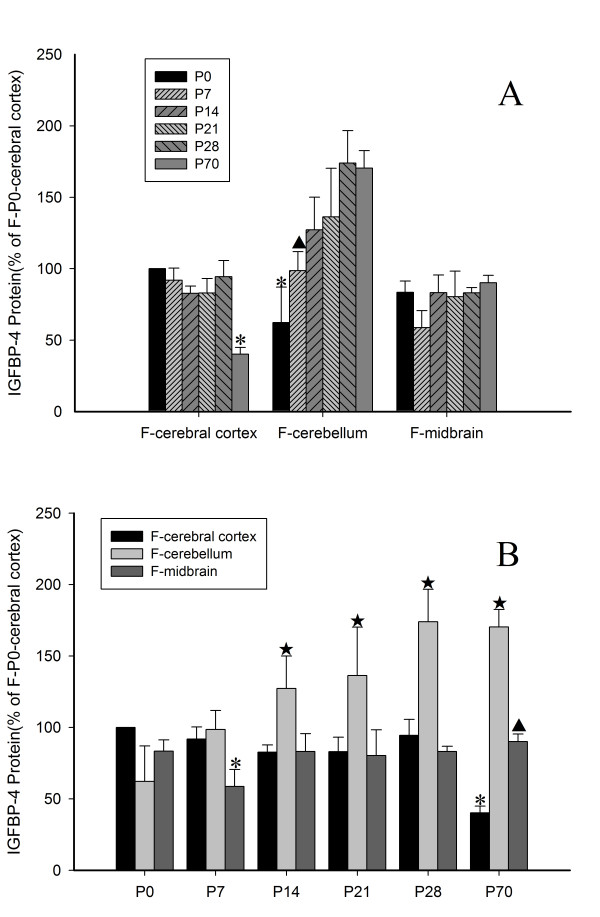
**IGFBP-4 protein expression in postnatal rat brains. (A)** The temporal expression of IGFBP-4 protein in specific brain regions. (For the cerebral cortex, *- The level of IGFBP-4 protein at P70 was significantly lower than at other time-points in the cerebral cortex. For the cerebellum, *- The level of IGFBP-4 protein in the cerebellum at P0 was significantly lower than at P21, P28, and P70; ▲- - The level of IGFBP-4 protein at P7 was significantly lower than that at P28 in the cerebellum. For the midbrain, there is not significant difference between different time-points. All *P* < 0.05) **(B)** The spatial expression of IGFBP-4 protein at specific time points. (At P0, there is not significant difference between different brain regions. At P7, *- the level of IGFBP-4 protein was lower in the midbrain than in the cerebral cortex and cerebellum. At P14, P21, and P28, ★- the IGFBP-4 protein in the cerebellum was higher than in the cerebral cortex and midbrain. At P70, *- the IGFBP-4 protein in the cerebral cortex was lower than in the cerebellum and midbrain; ▲- in midbrain is lower than in the cerebellum; ★- in the cerebellum was highest. All *P* < 0.05).

The amount of protein was determined densitometrically using Quantity One (Table 2). In the cerebral cortex, the level of IGFBP-4 protein showed little change in the early stages after birth (from P0 to P28), but it decreased significantly at P70. In the cerebellum, the level increased gradually from P0 to P28 and remained at a significantly high level at P70. In the midbrain, however, it remained relatively constant from P0 to P70 (Figure 7A).

**Table 2:** IGFBP-4 protein levels in the postnatal rat brains

**Protein**	**F-cerebral cortex**	**F-cerebellum**	**F-midbrain**
P0	100.00000	86.5417 ± 24.86347	83.4732 ± 7.86149
P7	91.9381 ± 8.42004	113.0409 ± 13.31736	58.7150 ± 11.85140
P14	82.7600 ± 5.03834	162.3909 ± 22.81005	83.2167 ± 12.41476
P21	83.0186 ± 10.16573	172.8639 ± 33.90615	80.3992 ± 17.94268
P28	94.4435 ± 11.20187	219.8978 ± 42.70306	83.1549 ± 3.677886
P70	40.1657 ± 4.74199	180.3804 ± 12.24069	90.1419 ± 5.22144

In order to emphasize the area specificity of IGFBP-4 protein expression in the brain, the differences between the cerebral cortex, the cerebellum and the midbrain at same time-points were further analyzed by using the same data as shown in Figure 7A. There was no significant difference in the level of IGFBP-4 protein among three brain regions, cortex, cerebellum, and midbrain, at P0. At P7, the level in the midbrain was significantly lower than that in the cortex and cerebellum (*p* < 0.05). At P14, P21, and P28, the cerebellum expressed a higher level of IGFBP-4 than did the cerebral cortex and the midbrain (*p* < 0.05). At P70, the expression of the protein remained at the highest level in the cerebellum, moderate in the midbrain, and lowest in the cortex (*p* < 0.05) (Figure 7B).

## Discussion

### Spatiotemperal expression patterns of IGFBP-4 in the rat brain

IGFBP-4 was initially purified from rat serum and human bone cell-conditioned medium in 1990 [18], and there have been several studies about IGFBP-4 expression during development. Transcripts for IGFBP-4 were detectable in the most mesodermally derived tissues of the mid- and late gestational mouse (E11-18) [13] and rat (E13-21) [14], as well as in the telencephalon and mesencephalon of the mid-gestational mouse (E11-13) [16]. IGFBP-4 expression is also easily detectable in the choroid plexus, meninges [15], cerebrum, olfactory bulb, cerebellum [16] in the E15 rat embryo, and the basal ganglia in the E20 rat embryo [15]. It is undetectable in the choroid plexus and meninges in the late gestational mouse fetus (E18) [13]. In the present study in the rat, IGFBP-4 expression was seen in the forebrain, midbrain, hindbrain, and also in the meningeal cells from E10.5, peaked at E13.5 to E14.5, and reached to its lowest level at E17.5 and E18.5. Our data have confirmed the findings thet IGFBP-4 expresses in ratforebrain (cerebrum, olfactory bulb, and basal ganglia) [15,16] and hindbrain (cerebellum) [16] at mid- and late gestation (E15, E20), and in the midbrain at E11-13 [13], but were inconsistent with the data that IGFBP-4 expression is not detectable in the rat brain at E14 [14] and neither in the mouse brain after E14 [13].

Postnatally, IGFBP-4 mRNA is found in the meningeal cell layer surrounding the developing cerebellum, the hippocampal formation [15], and the olfactory bulb [16,19]. In the adulthood, The expression of IGFBP-4 mRNA is detectable in the cerebral cortex (layers II and IV), olfactory peduncle (anterior olfactory nuclei), limbic system (hippocampus and amygdala), thalamus and basal ganglia, as well as choroid plexus and meninges [15].

The expression of IGFBP-4 in the postnatal cerebral cortex in present study using real time PCR is in part similar to that by Chernausek et al., using RNA blot hybridization [16], The mRNA level of IGFBP-4 is relatively low in the adult cerebral cortex, but shows a raising phase before P21. Whereas the protein level of IGFBP-4 remains relatively constant from P0 to P28. The different locations of IGFBP-4 between the mRNA and protein in the brain may be due to transportation of IGFBP-4 protein from its synthesized and secreted cells, where the IGFBP-4 mRNA is located, to the other regions.

In the caudate-putamen, IGFBP-4 expression displays a medial to lateral distribution gradient that is away from the neuroepithelium along the lateral wall of the lateral ventricles, the site of active neurogenesis [20]. This finding is similar to the ours that fluorescent signals of IGFBP-4 are not apparent in the cells near the ventricle from E16.5.

Our data showed a relatively lower level of IGFBP-4 mRNA in the cerebellum after birth, but a strikingly higher abundance of the protein during the first 4 postnatal weeks, compared with that in the cerebral cortex and midbrain. The level of IGFBP-4 protein increases gradually with the maturation of the postnatal cerebellum and remains at a high level until adulthood.

### The roles of IGFBP-4 in the brain development

Development of the brain starts early in the embryo and continues after birth. Growth factors usually have a wide variety of actions during the brain development, including survival, proliferation, differentiation, and migration of neural cells.

In the rat, neurogenesis, generation of neurons, begins from progenitor cells within ventricular zone (VZ) at E12, reaches a peak at E14 and stops at E18 when the subventricular zone (SVZ) continues to generate neurons. Glial cells are also produced in the SVZ at E18 [21-24]. Most of the astrocytes are generated during P0 to P2, and the generation of oligodendrocytes reaches a peak at P14 [25].

The separate timing of neurogenesis and gliogenesis in the brain has been described for many years, but the mechanisms underlying these changes in progenitor fate determination remain largely unknown. Some investigators believed that the mechanisms must include both changes in the intrinsic properties of neural progenitors and in their signaling environment [26]. Two ligands of the IGF system, IGF-I and IGF-II (especially IGF-I), have been shown to exert a wide variety of actions during development, promoting the brain growth, neuronal proliferation, and neuron number, while IGFBPs have inhibitory effects on the brain growth (IGFBP1, 2, 3, 6) and neuron number (IGFBP1) [27]. No reports are available on roles of IGFBP-4 in the brain development.

IGFBP-4 is known as a secreted peptide, and has been identified in almost all biological fluids [28]. IGFBP-4 expression is found to be selectively localized in mature differentiated neurons of the caudate-putamen [20]. Our earlier findings also indicated that IGFBP-4 expression was higher in mature neurons than in neural precursors [2]. In the present study, the temporal expression patterns of IGFBP-4 in the developing rat brain are coincident with the neurogenesis phases in the VZ and its expression is widespread in the forebrain other than the cells restricted near the ventricle at E16.5.

Formation of neuronal processes, formation and maturation of synapses in the cerebral cortex, are all postnatal events which occur mainly during the second and third postnatal weeks [29]. Since IGFBP-4 protein expression in the adult cortex is significantly lower than that during the first four weeks after birth, it can be speculated therefore, that IGFBP-4 may play a role in cortical neuron maturation. The neurons in the cerebellum emerge not only from the VZ before birth, but also from a secondary germinal zone, the external granular layer (EGL) after birth, with the maturation occurring at about 4 weeks of age [30,31]. It is reported that the immature EGL express IGF-1 receptor, whereas IGF-1 mRNA is reported in the Purkinje cells [32] and in the migrating, differentiating neurons of the developing cerebellum in the rat [33]. Thus, the replicating cells of the EGL are sandwiched between cells presumably secreting IGF-1 on one side and IGFBP-4 on the other. Our data have shown a less mRNA level of IGFBP-4 in the cerebellum than in the cerebral cortex but a strikingly higher abundance of protein level in the cerebellum from P14 to P70, suggesting that IGFBP-4 could play a role in the maintenance of cerebellar plasticity.

All of these findings, together with the data that IGFBP-4 has been shown to promote myocardial differentiation of embryonic stem cells [34] and adult mesenchymal stromal cells [35], suggest that IGFBP-4 can be quite strongly regarded as one important factor in those signaling environment for neurogenesis. It is reported that IGFBP-4 mRNA increases markedly in the ipsi-lateral cortex after hypoxic-ischemic insult [36], suggesting that IGFBP-4 may also play a role in neurogenesis during brain injury and repair.

Accumulating data strongly indicate that IGFBP-4 exerts inhibitory effects on IGF-promoted growth [37,38]. IGFBP-4 knockout mice are smaller than their wild-type littermates (7% less at E12.5, 16% at E14.5, and 11% at E16.5) [11]. Down-regulation of IGFBP-4 is associated with abnormal mitogenesis, such as in Wilms’ tumor [39]. Immunoelectron microscopy has revealed that IGFBP-4 is localized at the centrioles, and also it has a direct interaction with microtubules in primary astrocytes in the rat and human [40], as shown by an intracellular macromolecular complex of IGFBP-4 with four other proteins including MIZ-1 (a transcription factor that participates in the control of the cell cycle) [41]. MIZ-1 appears to be regulated by association with microtubules [42], indicating an involvement of IGFBP-4 in microtubule functions. Connexin-43, a junction protein, is known to interact directly with microtubules [43,44]. Overexpression of connexin-43 in C6 glioma cells results in decreased levels of IGFBP-3 and increased levels of IGFBP-4 and it may be responsible for the reduced proliferative capacity [45]. Interestingly, inhibition of IGFBP-4 expression in prostate cancer cells also lead to the suppression of cancer cell growth which may be caused by greatly increased levels of IGFBP-3 and -6 in these cells [46]. Therefore, it is reasonable to speculate that the IGF-independent effects of IGFBP-4 may involve the regulation of cell motility and proliferation through the instability of microtubule dynamics.

Certainly, the possible effects of IGFBP-4 in neurogenesis and proliferation during brain development mentioned above need to be further elucidated via gain-of-function and loss-of-function approaches and this is our ongoing research.

## Conclusion

The results of present study, together with other previous data, indicate that IGFBP-4 may have a significant role during CNS development, although some further research is required.

## Methods

### Animals

Embryonic and postnatal Sprague–Dawley rats at different developmental stages were used for the study. The day on which spermatozoa were found in the vaginal smear in the morning was defined as day 0.5 of pregnancy. Rat embryos on embryonic days E10.5, E11.5, E12.5, E13.5, E14.5, E15.5, E16.5, E17.5, and E18.5 were obtained by cesarean section. Eight brains from these embryos at each time-point were stored in -80°C for quantitative real-time PCR and Western blotting, and the other 8 brains were fixed with paraformaldehyde for immunohistochemistry (see below). Postnatal female rats (P0, P7, P14, P21, P28, and P70; 4 animals for each time-point) received an intraperitoneal injection of pentobarbital (50 mg/kg body weight). For each postnatal female rat, cerebral cortex, midbrain and cerebellum were separately dissected and stored in -80°C for quantitative real-time PCR and Western blotting. All procedures were approved by the Capital Medical University Animal Care Committee in accordance with the policies established in the Chinese Guide to Care and Use of Experimental Animals. The number of animals used and their suffering was minimized.

### Immunohistochemistry

Brains were fixed by immersion (for the embryos at days E10.5–E15.5), or by transcardial perfusion (for those at day E16.5 or older) with 4% paraformaldehyde in phosphate buffered saline (PBS, pH 7.4). Brains were immersed in the same fixative at 4°C overnight and then transferred to a solution of 20% sucrose in PBS (pH 7.4) for cryoprotection at 4°C overnight again, and subsequently frozen in Tissue Tek O.C.T. compound (Miles, Inc., Elkhart, IN). Sagittal cryostat sections, 18 μm-thick, were cut and collected onto poly-L-lysine-coated slides. Sections on glass slides were treated with heat in citrate buffer (10 mM, pH 6.0) at 95°C for 5 min. Then the sections were treated with 0.1% hydrogen peroxide in PBS containing 0.1% Triton X-100 (PBST) for 30 min to block endogenous peroxidase activity. After incubation with 10% normal goat serum, the sections were incubated overnight with goat anti-IGFBP-4 (C-20) (1:50; Santa Cruz Biotechnology, sc-6009). The immunostaining for IGFBP-4 expression was validated by using rabbit anti-IGFBP4 (H-85) (Santa Cruz, sc-13092). The normal goat or rabbit serum was used to replace anti-IGFBP-4 for negative control purpose. The sections were washed with PBST 4 times, and then incubated for 60 min with a species-specific secondary antibody conjugated to AlexaFluor 488 (1:1000; Molecular Probes) for fluorescent detection. All sections were counterstained with Hoechst33342 (0.1 μg/ml, 10 min) to visualize cell nuclei. After staining, the samples were examined with a Leica DM 4000B microscope (Leica, Germany). All immunocytochemical experiments were repeated twice for confirmation.

### RNA isolation and reverse transcription

Total RNA was extracted from rat embryo brains (n = 4 each time-point) from days E10.5 to E18.5 and P0, using TRIzol reagent (Invitrogen). For postnatal rats, cerebral cortex, midbrain, and cerebellum were separately dissected and total RNA was extracted (4 brains for each time-point). The integrity and purification of RNA samples were monitored by formaldehyde/agarose gel electrophoresis, and RNA concentrations were determined by optical density measurements at 260 nm. The cDNA was synthesized using 2 μg of total RNA from each embryonic brain using Superscript and oligo(dT) (Invitrogen), and used in accordance with the manufacturer’s instructions. Two separate negative-control reactions, either without RNA or without SuperScript, were carried out with each reaction.

### Quantitative real-time polymerase chain reaction (real-time PCR)

PCR was performed as described by the manufacturer using the Power SYBR Green PCR Master Mix (Applied Biosystems, 4367649). The final reaction contained 10 μl SYBR green/enzyme reaction mix, 0.5 μM primer and 1 μl of cDNA, in a total volume of 20 μl PCR conditions were 50°C for 2 min, 95°C for 10 min, followed by 40 cycles of 95°C for 15 sec, and 60°C for 1 min. Melting curve analysis was applied to all reactions to ensure homogeneity of the amplification products. The product size for each primer set was confirmed by gel-electrophoresis. Potential contamination was monitored by using non-template controls as references. Sequence detection software version 1.2 was used for all data analysis. mRNA levels were normalized using β-actin as housekeeping gene and compared with levels in P0. All results were repeated in 6 to 8 independent experiments and performed in triplicate each time and were expressed as mean ± SEM. The primers for IGFBP-4 are as follows: forward: CAGAGCCGTACCCACGAAGA, reverse: CCGTTGCGGTCACAGTTG.

### Western blotting

The whole brains (n = 4 each time-point) dissected from embryos at days E10.5 to E18.5 and P0 were homogenized in cold RIPA buffer (Roche). For postnatal rats, three areas of the brain, cerebral cortex, midbrain, and cerebellum, were separately dissected and homogenized in cold RIPA buffer (n = 4 each time-point). Protein concentration was determined using the BCA protein assay reagent (Pierce Biotechnology). Equal amounts of total cellular protein were denatured at 100°C for 10 min. Each lane in the separating gel contained approximately 30 mg of total protein which was separated on 12% SDS polyacrylamide gels and transferred to PVDF membranes. After transferring the proteins onto the membranes (Bio-Rad), the membranes were blocked in 5% skim milk for 1 hr and, incubated with goat anti-IGFBP-4 antibody (H-85) (1:200; Santa Cruz Biotechnology, sc-13092) overnight at 4°C. Antibody binding localization was visualized by incubation with rabbit anti-goat HRP-conjugated secondary antibody (1:10,000, Golden Bridge Biotechnology) on the following day. Chemiluminescence detection was performed with an ECL kit (Pierce, Rockford, IL). The amount of protein was determined densitometrically using Quantity One software (Gel Doc 2000 Imagine System, Bio-Rad Inc., USA). All western blot experiments were repeated at least three times.

### Statistical analysis

Quantitative experiments were repeated in at least 4 independent experiments, and the mean value was used for statistical analysis. Results were presented as means ± standard error (SE). The significance of differences in mRNA or protein levels of IGFBP4 in the brain at different time-points was determined using one-way ANOVA by SPSS. A value of p < 0.05 was considered statistically significant.

## Competing interests

The authors declare that they have no competing interests.

## Authors’ contributions

XHJ carried out Immunohistochemistry, RNA isolation and reverse transcription, Quantitative real-time polymerase chain reaction displayed in this manuscript, and drafted the manuscript. JPZ and LLJ did the western blot analysis. YJL fed animals and CLX perfused the rats. BBW designed part of the real time polymerase chain reaction experiments. XFZ designed part of the immunohistochemical experiments. QYX conceived the study and helped drafting the manuscript. All authors read and approved the final manuscript.

## Supplementary Material

Additional file 1 The analysis results of IGFBP-4 mRNA at P0. Click here for file
