# Supplementary material for: Temporal expression patterns of insulin-like growth factor binding protein-4 in the embryonic and postnatal rat brain
Source: BMC Neurosci. 2013 Oct 31;14:132. doi: 10.1186/1471-2202-14-132 (PMC3871010; doi:10.1186/1471-2202-14-132)
Supplement: Additional file 1 — The analysis results of IGFBP-4 mRNA at P0. [file 1471-2202-14-132-S1.pdf]

<!--Text used as the document title (displayed in the title bar).-->

Multiple Comparisons

Dependent Variable: RQ of IGFBP4 mRNA

			Mean Difference (I-J)	Std. Error	Sig.	95% Confidence Interval	
	(I) 样本号	(J) 样本号				Lower Bound	Upper Bound
LSD	P0-F-cortex	P0-F-cerebellum	-.01782(*)	.002102	.000	-.02215	-.01348
		P0-F-midbrain	-.09569(*)	.002102	.000	-.10003	-.09136
	P0-F-cerebellum	P0-F-cortex	.01782(*)	.002102	.000	.01348	.02215
		P0-F-midbrain	-.07788(*)	.002102	.000	-.08221	-.07354
	P0-F-midbrain	P0-F-cortex	.09569(*)	.002102	.000	.09136	.10003
		P0-F-cerebellum	.07788(*)	.002102	.000	.07354	.08221
* The mean difference is significant at the .05 level.							

Homogeneous Subsets

RQ of IGFBP4 mRNA

		N	Subset for alpha = .05		
	样本号		1	2	3
Student-Newman-Keuls(a)	P0-F-cortex	9	1.00000		
	P0-F-cerebellum	9		1.01782	
	P0-F-midbrain	9			1.09569
	Sig.		1.000	1.000	1.000
Means for groups in homogeneous subsets are displayed.					
a Uses Harmonic Mean Sample Size = 9.000.					

[illegible]
